# AI driven fault diagnosis approach for stator turn to turn faults in induction motors

**DOI:** 10.1038/s41598-025-04462-x

**Published:** 2025-06-20

**Authors:** Tamer A. Kawady, Wagdy M. Khater, Hagar G. Abu-Faty, Mohamed A. Izzularab, Mohamed E. Ibrahim

**Affiliations:** 1 Electrical Engineering Department, Faculty of Engineering, Menoufia University, Shebin El Kom, 32511 Egypt; 2 Dredging Department, Suez Canal Authority, Ismailia, Egypt; 3 Data Science Department, Faculty of Artificial Intelligence, Menoufia University, Shebin El Kom, Egypt; 4 High Voltage and Dielectric Materials Lab., Faculty of Engineering, Menoufia University, Shebin El Kom, 32511 Egypt; 5 Faculty of Technological Industry and Energy, Delta Technological University, Quweisna, Egypt

**Keywords:** Induction motor, Fault diagnosis, Inter-turn fault (ITF), Machine learning (ML), Discrete Fourier transform (DFT), AI-based fault detection, Electrical and electronic engineering, Computer science

## Abstract

Induction motors (IMs) are vital in industrial applications. Although all motor faults can disrupt its operation significantly, stator turn to turn faults (ITFs) are the most challenging one due to their detection difficulties. This paper introduces an AI-based approach to detect ITFs and assess their severity. A simulation based on an accurate mathematical model of the IM under ITFs is employed to generate the training data. Recognizing that ITFs directly affect the motor’s current balance, complex current unbalance coefficient is identified and used as the key feature for detecting ITFs. Since unbalanced supply voltage (USV) can also disrupt current balance, the AI models are trained to account for USV by incorporating complex voltage unbalance coefficient that helps to distinguish between ITF-induced and voltage-induced imbalances. After feature extraction, the AI models are trained and validated with simulation data. The approach’s effectiveness is further tested using an experimental setup, where measurements from motors under various fault conditions, including USV scenarios, are considered. The results indicate that the gradient boosting model outperforms other ML models in detecting ITFs in IMs and assessing their severity. In the pursuit of achieving highest possible performance, DNN is tested and compared with ML models. The study reveals that DNN demonstrates superior performance in all tested scenarios including USV making DNN the top performer that to be used in the proposed approach. The proposed AI-based approach based on DNN offers high accuracy in fault detection and can effectively distinguish between ITFs and USV-induced anomalies, maintaining low estimation errors and robust performance across different operational conditions.

## Introduction

Induction motors (IMs) are considered the most important and widely used electric motors in almost all industrial applications due to their efficiency, durability, low cost, and low maintenance requirements. They play a fundamental role in numerous sectors, including manufacturing, transportation, and household appliances, making them indispensable in modern industry. IMs are often equipped with power electronic converters to address speed control issues, further solidifying their status as the backbone of industrial applications^1^. Despite their robustness, IMs are vulnerable to various mechanical and electrical faults caused by electrical, thermal, and mechanical stresses, which can disrupt normal operations, leading to significant financial losses, reduced productivity, and costly emergency maintenance^2^. According to fault statistics, internal electrical faults in IMs include approximately 10% for rotor faults and around 37% for stator faults. Among the different types of faults, inter-turn faults (ITFs) represent a significant portion, accounting for over 36% of motor stator failures^3^. ITFs are critical as they can rapidly progress to severe damage, necessitating early detection to prevent unexpected breakdowns and ensure system reliability. Consequently, developing effective ITFs detection techniques for IM is essential for modern industry, to avoid potential damage, reduce downtimes, and extend the operating lifetimes of these motors.

There are three primary approaches for diagnosing ITFs in IMs: model-based methods, signal analysis-based methods, and artificial intelligence (AI)-based methods. Model-based methods involve creating a mathematical model of the motor and comparing its expected behavior with the actual behavior to identify discrepancies indicative of faults^4–9^. Model-based methods’ primary advantages include high accuracy, adaptability to changing conditions, and effective fault isolation. However, they also come with limitations such as the need for precise parameter tuning, computational intensity, implementation complexity, and reliance on detailed motor parameters.

Various signal analysis-based methods have been developed to address the challenges associated with model-based diagnosis techniques. These methods analyze specific characteristics of electrical signals to identify abnormalities that indicate the presence of faults. Babaa and Bennis^4^ highlight the importance of motor current signature analysis (MCSA) and discrete wavelet transform (DWT) for identifying frequency shifts in the stator current under fault conditions. Similarly, Casimir et al.^10^ utilized MCSA in conjunction with harmonic analysis to diagnose stator winding faults, including ITFs. Mejia-Barron et al.^11^ emphasized the potential of MCSA for detecting ITFs by analyzing current waveforms and using multilayer neural network-based model. Ukil^12^ introduced a method for detecting ITFs using zero crossing instant analysis, despite its reduced effectiveness under noisy conditions or varying load profiles. Both Fortescue transformation and Park’s vector analysis were utilized for fault detection as seen in^2,13,14^. Maraaba et al.^15^ explored acoustic signal analysis for diagnosing faults, leveraging non-invasive monitoring to detect a wide range of faults, but this approach can be susceptible to ambient noise and requires sophisticated signal processing. Choudhary et al.^16^ recently proposed a vibro-acoustic fusion method using MI-CNN and CQ-NSGT to convert vibration and acoustic signals into time–frequency spectra for accurate fault detection in IMs and other rotating machinery under varying conditions. Eltabach^17^ focused on instantaneous power analysis, identifying deviations in power consumption patterns to detect faults, though it demands high sampling rates and may be affected by power supply fluctuations. Thermal imaging has been effectively utilized in fault detection for electric motors, as demonstrated in^18–20^. These studies all employ the principle of identifying hot spots, which serve as early indicators of faults. Additionally, AlShorman et al.^21^ reviewed recent advances in image-based intelligent techniques for induction motor fault detection, emphasizing the effectiveness of thermal imaging for non-contact, real-time monitoring, despite its reliance on specialized equipment and sensitivity to environmental conditions. In summary, signal analysis-based methods offer several advantages, including non-invasive monitoring, real-time applicability, and the ability to detect a wide range of faults. However, these methods also come with limitations such as susceptibility to noise, high computational requirements, and the need for specialized equipment.

In recent years, AI techniques, including machine learning (ML), neural networks, fuzzy logic, and genetic algorithms, have become essential for accurately diagnosing faults in IMs, offering high precision and adaptability to meet the increasing complexity and reliability demands of modern industrial systems. AI-based diagnostic tools provide the necessary sophistication and accuracy, making them indispensable in the maintenance and monitoring of these critical machines^22^. Rehman et al.^23^ introduced an AI-based current and flux correlation analysis technique, providing high accuracy for stator fault detection directed to doubly-fed induction generators. In^24^ ML approach utilizing neural networks was employed to classify fault conditions from stator current signals, achieving high accuracy and highlighting the effectiveness of AI in early fault detection. Filippetti et al.^25,26^ reviewed recent advancements in AI-based fault diagnosis for IM drives, highlighting the effectiveness of various techniques in identifying a range of motor faults. Siddique et al.^27^ presented a comprehensive review of AI applications, particularly neural networks and fuzzy logic, for diagnosing stator faults in induction machines, assessing both their performance and limitations.

Bouzid et al.^28^ presented a neural network-based approach for automatically locating stator ITFs in IMs, validated through simulations and experimental tests with high accuracy. Benbouzid^29^ reviewed signature analysis techniques, such as current and vibration analysis, for detecting faults in IMs. Mohamed^3^ investigated AI-based approaches, including neural networks and ML algorithms, for fault diagnosis in IMs, supporting their effectiveness with experimental validation. Recently, Vo et al.^30^ proposed a deep learning model combining 1D-CNN and RNN pipeline, utilizing attention mechanisms to extract spatial and temporal features from raw electrical signals. This approach enhances fault detection accuracy and streamlines the diagnostic process, demonstrating strong potential for real-time industrial applications. In^31^, fault detection is proposed using deep learning techniques such as CNN and LSTMs, achieving excellent accuracy in classifying and localizing faults. Sandeep et al.^32^ successfully detected external faults using a multi-class extreme learning machine neural network (ELM-NN) with excellent accuracy. Yaw et al.^33^ proposed a method combining clustering artificial neural networks (ANNs) with particle swarm optimization (PSO) to locate and assess the severity of ITFs, though the model’s complexity led to variable results. Chikkam et al.^34^ estimated fault severity using features extracted from DWT of current signals, showing higher accuracy for ANN compared to other ML tools. Moosavi et al.^35^ employed analytical and finite element models to train an MLP for estimating stator winding shorted turns, demonstrating effectiveness with accuracy ranges between 88 and 99%. Bazan et al.^36^ used MLP and RBFN for detecting ITFs faults under unbalanced voltage with accuracy between 93 and 99%. Nabunita et al.^37^ computed a coefficient for MLP, achieving 99.6% accuracy for ITFs fault detection.

In summary, AI-based methods provide notable benefits such as high diagnostic accuracy, adaptability to varying operational conditions, and efficient fault isolation. However, they also present certain challenges, including the need for careful parameter tuning, reliance on large volumes of labeled training data to achieve reliable performance, and potential issues with generalization to unseen fault scenarios. While advancements in computing power have mitigated some of the computational demands, ensuring robust and transferable models remains a critical concern.

Unbalanced supply voltage (USV) presents a major challenge in fault diagnosis, as it can cause false alarms and obscure the presence of ITFs in IMs. Accurate differentiation between USV-related disturbances and actual winding faults is essential, yet difficult due to their similar impact on motor behavior. While some studies have employed negative sequence voltage^38^, negative sequence impedance^39^, and third harmonic analysis of the FFT current signal^15^ to detect or distinguish USV from ITFs, such methods remain limited. The effectiveness of ITFs detection still heavily depends on the fault’s severity and location, and the presence of USV can mask or mimic ITFs symptoms. This diagnostic confusion presents a critical gap in the current literature, many existing methods do not provide sufficient mechanisms to distinguish between current imbalance caused by ITFs and that caused by USV. As a result, there is a strong need for a focused, feature-driven approach that can accurately isolate the true fault source.

In response to this challenge, this study proposes a novel diagnostic approach based on various AI techniques to enhance the detection and severity estimation of ITFs in IMs. The proposed approach aims to improve diagnostic accuracy, reduce false alarms, and ensure the reliable operation of induction motors. Its key innovation lies in its ability to detect ITFs even in the presence of USV, achieved by extracting two distinct features from current and voltage signals at the motor terminals. The effectiveness of the proposed method has been experimentally validated, demonstrating its robustness under realistic operating conditions. In the following sections, the design and implementation of the proposed diagnostic approach will be detailed, starting with the modeling of fault conditions and feature extraction, followed by the AI training process and experimental validation results that demonstrate the system’s effectiveness under various operating conditions.

## Outline of the proposed ITFs diagnostic approach

The primary objective of this research is to employ AI techniques, including various ML algorithms and deep learning (DNN), for the detection of ITFs in IMs, particularly under conditions of USV. The study aims not only to estimate the severity of ITFs by determining their percentage but also to address the critical challenge of differentiating ITFs from USV-induced anomalies, which are often misclassified by traditional diagnostic systems and lead to false alarms. To meet this challenge, the proposed approach relies on two carefully engineered features, the Complex Current Unbalance Coefficient (δ_c_) and the complex voltage unbalance coefficient (δ_v_), which together provide a robust basis for distinguishing between ITFs and external power supply disturbances. Both ML and DNN models were applied to these features to assess which method yields better diagnostic accuracy and generalizability.

**Fig. 1:**
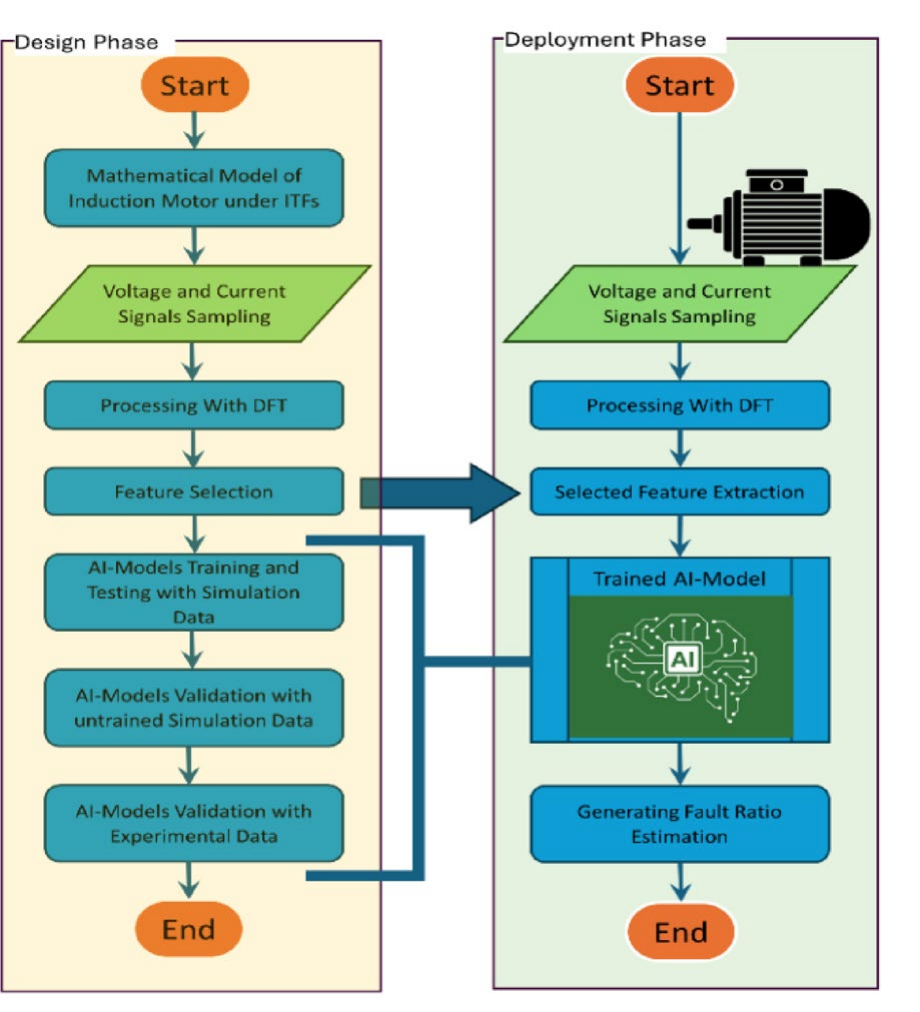
Flow chart of the proposed ITFs diagnostic.

By comparing the diagnostic performance of these models, this research identifies the most effective AI-based strategy for reliable fault detection and severity estimation under real-world operational conditions. The proposed diagnostic system is systematically structured into two key phases: the design phase and the deployment phase, as illustrated in Fig. 1. This methodology ensures both rigorous development and practical applicability for industrial use.

The diagnostic process commences with the design phase. Initially, a mathematical model of the IMs, incorporating ITFs, is utilized^40^. This model simulates the behavior of the motor under fault conditions and serves as the basis for feature extraction and AI training. From this simulation, voltage and current signals are sampled and processed using the discrete Fourier transform (DFT) to obtain power frequency phasors, enabling the extraction of fault-relevant features. The next step involves the selection of relevant features from the processed signals, which are critical for effective fault detection and diagnosis.

These features are then used to train various ML algorithms, equipping them with the capability to recognize fault patterns. Validation is performed using untrained simulation data to evaluate the generalization ability of the models. In parallel, DNN undergo the same training and validation process. A comparative analysis of ML and DNN models is then conducted to identify the most accurate and generalizable diagnostic approach.

To assess robustness in practical scenarios, the best-performing models are validated using experimental data. This final validation step ensures that the models are capable of accurate fault detection and severity estimation under real-world operating conditions. Ultimately, the performance is compared for identifying the most effective model for reliable fault detection and severity estimation. Successful completion of these tasks concludes the design phase and confirms the system’s readiness for practical application.

The deployment phase involves the real-time operation of the trained diagnostic system. Real-time voltage and current signals are sampled from the motor and transformed using DFT to extract phasors. The same feature extraction procedure is applied, and the resulting features are fed into the trained AI model, which estimates the presence and severity of faults based on learned patterns.

The system outputs a fault ratio, quantifying the degree of abnormality. A threshold of 1% is defined: ratios below this value indicate normal operation, while higher values signify the presence of ITFs. To prevent false alarms caused by transient disturbances or noise, a fault is only confirmed if the threshold is exceeded across three consecutive cycles. This criterion enhances the reliability of the system in industrial environments.

By integrating advanced signal processing with AI-based diagnostics, the proposed approach offers a robust and effective method for ITF detection and severity estimation in IMs. The model-based design and real-time implementation ensure both high diagnostic accuracy and industrial applicability, marking a significant contribution to the field of IMs diagnostics.

The subsequent sections will provide a detailed explanation of each component of the diagnostic system, covering its development, validation, and implementation.

## Inter-turn-fault induction motor model

In this study, detailed modeling of IMs faults that facilitating the incorporating stator ITFs modeling was utilized based on the motor parameters listed in Table 1. This model has been developed, and its accuracy has been confirmed through simulation and experimental verification as detailed in^40^. These simulations were specifically designed to cover a broad spectrum of fault ratios under varying conditions of voltage imbalance, thereby enabling a comprehensive analysis of potential diagnostic markers.

**Table 1:** Selected motor parameters for simulation tests.

Rated power	4 Hp
Rated voltage	400 V/Star
Rated current	5.3 A
Frequency F	50 Hz
Number of poles P	4
Stator resistance R_s_	4.533 Ω
Rotor resistance R_r_	4.121 Ω
Stator leakage inductance ${L}_{{\ell}s}$	0.0170 H
Rotor leakage inductance ${L}_{{\ell}r}$	0.0170 H
Magnetizing inductance ${L}_{m}$	0.2497 H
No-load resistance R_m_	987.023 Ω
Moment of inertia J	0.018750 kg m^2^

**Fig. 2:**
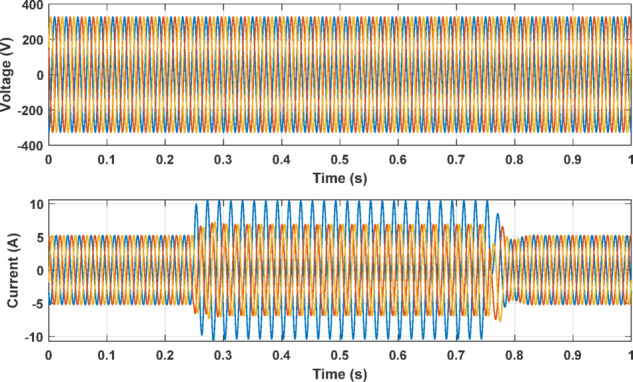
Supply voltage and motor current of simulation with 15% ITF.

Through computational analysis of IM model simulations, it has been observed that ITFs lead to an increase in the current’s amplitude within the affected phase. Conversely, in scenarios of USV affecting a single phase, there’s a noticeable decrease in current amplitude, while the current in unaffected phases experiences an increase. The manifestation of ITFs in the motor invariably results in a discernible current imbalance, which serves as a quantifiable measure of the fault’s severity. On the other hand, supply voltage imbalances create a current imbalance effect similar to ITFs, potentially leading to diagnostic confusion. Figure 2 displays the outcome of a simulated scenario in which 15% of the windings in phase A were shorted. The figure illustrates a significant increase in the current of the defective phase as compared with other ones. Additionally, a current imbalance was observed, as a consequence of the fault in the system.

**Fig. 3:**
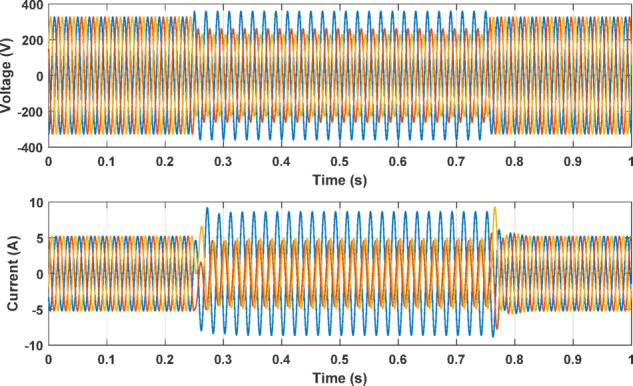
Motor performance with 10% increase in phase A voltage with voltage unbalance of phases B and C.

Conversely, Fig. 3 presents a different simulation scenario, where phase A’s voltage was increased by 10%, while phase B and C encountered a decrement of 20% and 30%, respectively. This supply voltage unbalance leads to an elevation in the current of phase A and disrupts the balance of current among the three phases. It is essential to note that, within this specific simulation, the motor remains healthy with no ITF condition. However, the latter case may be unfortunately recognized as an ITF. Hence, the supply voltage unbalance can greatly influence the results of ITF detection schemes.

In the pursuit of identifying optimal indicators for the proposed diagnostic methodology, simulations of this model were conducted to assess both current and voltage unbalance factors. The analysis of these factors is crucial as they form the basis for the subsequent section’s discussion, aiming to refine the accuracy of fault detection approach. This step is instrumental in distinguishing between the effects of ITFs and USV, ensuring a reliable and precise fault diagnosis in IMs.

## Fault features analysis

To measure and quantify the unbalance in the induction motor, it is essential first to calculate the voltage and current phasors. The accurate tracking of these phasors is crucial for determining the sequence components, which are then used to assess the unbalance. The voltage and current phasors are obtained using a recursive DFT^41^. Once the voltage and current phasors are obtained, the sequence components can be calculated.

### Sequence components-based current unbalance coefficient

Using the obtained sequence component phasors, the unbalance in the motor’s drawn current can be easily calculated from Eq. (1), defining factor *δ*_*c*_*,* which represents the complex current unbalance coefficient as.

1 $$\delta_{c} = \frac{{I_{N} \left| \!{\underline {\, {\theta_{N} } \,}} \right. - I_{0} \left| \!{\underline {\, {\theta_{0} } \,}} \right. }}{{I_{p} \left| \!{\underline {\, {\theta_{p} } \,}} \right. }}$$

where $I_{P}$, *I*_*N*_, *I*_0_ are the positive, negative and zero components of the current respectively. While $\theta_{P}$, *θ*_*N*_, *θ*_0_ are the displacement angle of positive, negative and zero components of the current respectively.

The absolute value of the coefficient *δ*_*c*_ is used as measure of the current unbalance introduced by the presence of ITFs in the stator windings. To explore the effect of faulted ratio *k*_*i*_ on the unbalance factor *δ*_*c*_, a set of fault ratios is simulated and for each case the value of *δ*_*c*_ is calculated.

**Fig. 4:**
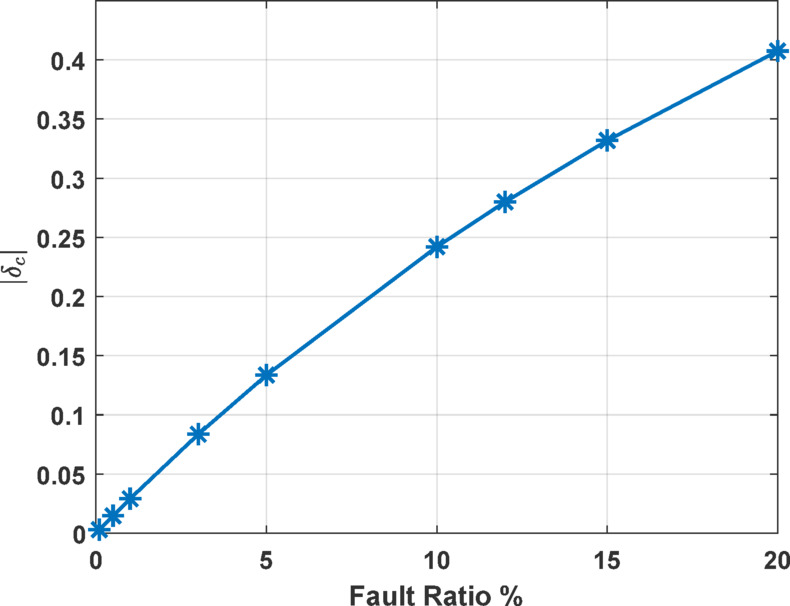
Coefficient *δ*_*c*_ evolution as function of ITF percentages.

Figure 4 summarizes the results obtained at different fault ratios. The figure illustrates a clear positive correlation: as the fault ratio *k*_*i*_ increases, the value of *δ*_*c*_ also increases linearly. This suggests that higher percentages of ITFs in the stator windings contribute to a greater degree of current unbalance within the system. Each data point on the graph corresponds to a simulated condition with a specific fault ratio, demonstrating how *δ*_*c*_ escalates consistently with the severity of the fault. This trend underscores the efficacy of *δ*_*c*_ as a diagnostic metric for assessing ITFs fault severity within the induction motor.

**Fig. 5:**
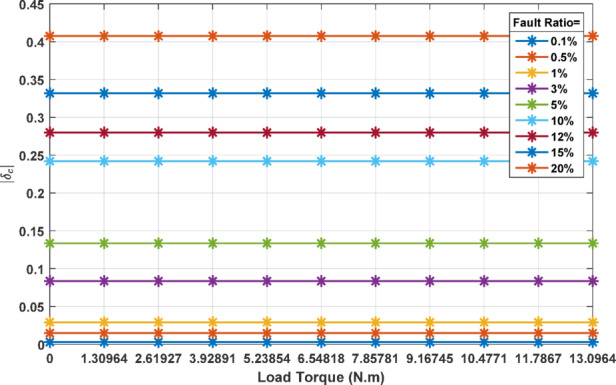
Coefficient *δ*_*c*_ evolution as function of motor loading.

Figure 5 explores the dynamics between the current unbalance factor *δ*_*c*_, and the motor’s loading conditions. In this representation, different lines are plotted for each fault ratio, illustrating the behavior of *δ*_*c*_ across a spectrum of load scenarios. Notably, the value of *δ*_*c*_ remains constant regardless of changes in the motor load. This consistency implies that the impact of ITFs on current unbalance is independent of the motor’s operational load, reinforcing using *δ*_*c*_ as a robust indicator for detecting ITFs and their respective severity. The invariance of *δ*_*c*_ with respect to load conditions simplifies fault diagnosis, as the factor can be considered reliable and unaffected by the motor’s load variations, making it a dependable tool for monitoring motor health.

### Sequence components-based voltage unbalance coefficient

**Fig. 6:**
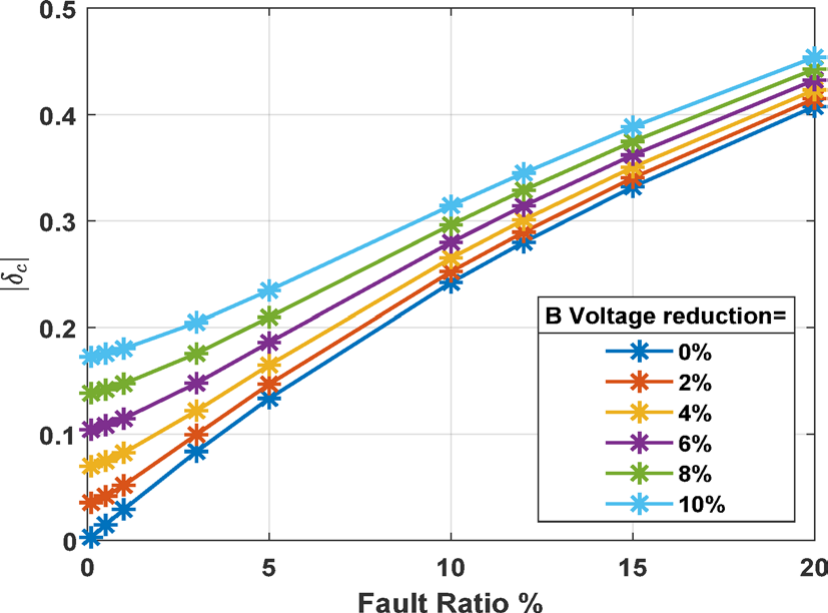
Impact of voltage unbalance on current unbalance coefficient *δ*_*c*_.

Although *δ*_*c*_ is robust in indicating the presence and severity of ITFs in stator windings, it is also influenced by other factors, most notably, USV. Figure 6 illustrates the influence of varying voltage reduction percentages, as USV condition, on the current unbalance factor *δ*_*c*_ across different fault ratios. This depiction is crucial for understanding the diagnostic challenges associated with using *δ*_*c*_ as indicator of ITFs in stator windings. From Fig. 6, a consistent linear increase is observed in *δ*_*c*_ as the fault ratio increases for all levels of voltage reduction, reinforcing *δ*_*c*_ as a sensitive indicator of fault severity under stable voltage conditions. There is a noticeable trend where *δ*_*c*_ values increase with the percentage of voltage reduction. For instance, at a 20% fault ratio, *δ*_*c*_ ranges from approximately 0.4 with no voltage reduction to about 0.46 at a 10% voltage reduction. This trend indicates that voltage unbalance aggravates the calculated current unbalance, potentially leading to overestimations of fault severity.

Complex voltage unbalance coefficient *δ*_*v*_ is essential for assessing the health and efficiency of IMs under varying electrical supply conditions. Defined by Eq. (2) using the sequence components of the supply voltage, *δ*_*v*_ provides a quantifiable measure of voltage asymmetry in the motor’s supply.

2 $$\delta_{V} = \frac{{V_{N} \left| \!{\underline {\, {\varphi_{N} } \,}} \right. - V_{0} \left| \!{\underline {\, {\varphi_{0} } \,}} \right. }}{{V_{p} \left| \!{\underline {\, {\varphi_{p} } \,}} \right. }}$$

where $V_{P}$, *V*_*N*_,* V*_0_ are the positive, negative and zero components of the supply voltage respectively. While $\varphi_{P}$,* φ*_*N*_,* φ*_0_ are the displacement angle of positive, negative and zero components of the voltage respectively.

Incorporating the complex value of *δ*_*v*_ is an effective strategy for covering all possible scenarios of USV. By utilizing the complex nature of *δ*_*v* _, the analysis not only accounts for the magnitude of voltage imbalances but also captures the phase angle differences, providing a comprehensive overview of the voltage conditions impacting the motor.

## AI models for ITFs diagnosis

In recent years, there has been an increasing reliance on advanced AI techniques to address complex challenges across various domains. The ability to automatically extract relevant features from raw data, without extensive manual intervention, enhances the accuracy and scalability of diagnostic tasks. The demonstrated effectiveness of these AI approaches in benchmark studies underscores their superiority over traditional methods, reinforcing their pivotal role in advancing modern fault diagnosis technologies.

Nine ML models were employed for the fault diagnosis system, each carefully selected based on its unique strengths in handling different aspects of data. These models; K-nearest neighbors (kNN), gradient boosting, random forest (RF), AdaBoost, decision tree, support vector machine (SVM), linear regression, partial least squares (PLS), and stochastic gradient descent (SGD), represent the majority of commonly used ML techniques for fault diagnosis in IMs, as widely reported in the literature^42–47^.

### Training and testing of ML-models

The selected ML models were trained and tested offline to ensure robust performance across various operational conditions. To achieve a highly generalized algorithm, a dataset was prepared, encompassing comprehensive variations in fault occurrences and supply voltages. Specifically, the dataset included 22,218 cases, covering a range of fault ratios from 0.2 to 1.0% in increments of 0.2%, and from 2 to 73% in increments of 0.4% for balanced supply scenarios. Additionally, same fault cases were considered for reductions in phase C voltage from 1 to 10%, along with corresponding reductions in phase B for each phase C reduction point, were included.

The implementation of selected ML models was carried out using Orange Data Mining (version 3.37.0). Orange is a versatile open-source data mining software known for its user-friendly interface and ability to handle a wide range of ML tasks. It provides an effective platform for developing, testing, and evaluating the models used in this study. In the configuration of these models within Orange, specific parameters were selected to optimize each model’s utility for fault diagnosis. AdaBoost was configured with tree as the base estimator, using 60 estimators and a learning rate of 1.0, employing the SAMME.R algorithm. Gradient Boosting was applied using the xgboost method, set with 150 trees, a learning rate of 0.3, a maximum depth of 6 for individual trees, and a regularization lambda of 1. For kNN, the model was set up with 5 neighbors and used the Manhattan metric, weighting distances between instances. Linear regression was implemented to fit the intercept, utilizing ridge regularization with an alpha of 0.0001. PLS was adjusted with 2 components, and an iteration limit of 500. RF employed 150 trees, limited to a depth of 3, with a minimum of 5 instances per node. SGD utilized a squared loss function, with a constant learning rate of 0.01 and ridge regularization at a strength of 0.00001. SVM was set with an RBF kernel, a cost parameter of 1.0. Finally, the decision tree was configured to limit the maximum tree depth to 100, with a minimum of 3 instances in leaves and 5 for a split, designed to stop when the majority classification reached 95%.

Following the configuration of each ML model, their effectiveness in diagnosing faults was tested. To measure the performance of these ML models against untrained data, a 10-fold cross-validation was conducted, which provides a robust estimate of the model’s ability to generalize to new datasets. The performance metrics for each model, including mean squared error (MSE), root mean squared error (RMSE), mean absolute error (MAE), and the coefficient of determination (R^2^), are summarized in Table 2.

**Table 2:** ML-models performance metrics for 10-fold cross validation.

Model	MSE	RMSE	MAE	R^2^
Gradient boosting	0.084	0.289	0.198	1
kNN	0.259	0.509	0.23	1
AdaBoost	0.309	0.556	0.177	0.999
Random forest	0.394	0.628	0.343	0.999
Tree	0.771	0.878	0.38	0.999
Linear regression	35.78	5.981	5.172	0.934
Stochastic gradient descent	36.52	6.043	5.201	0.933
PLS	37.71	6.141	5.283	0.93
SVM	548.5	23.421	20.39	− 0.012

The cross-validation results indicated that Gradient Boosting emerged as the top performer, achieving the lowest MSE (0.084) and an R^2^ score of 1, which suggests that it captures the variance in the data with near-perfect accuracy. kNN and RF models also showed strong performance, though with slightly higher MSE values, indicating their effectiveness but slightly less accurate compared to Gradient Boosting. AdaBoost and Decision Tree exhibited moderate performance; while AdaBoost struck a reasonable balance between error metrics and achieved a high R^2^ score, decision tree had the highest MSE and RMSE values, reflecting greater prediction errors. On the other hand, linear regression, SGD and PLS performed relatively poorly, with higher error metrics and R^2^ values slightly below 1, indicating their limitations in capturing the complex patterns in the dataset. Notably, SVM model was the least effective, with a negative R^2^ value, signifying its failure to fit the data appropriately, performing worse than a simple mean predictor.

To achieve the highest possible performance in detecting ITFs within IMs and accurately assessing their severity, the exploration of DNNs has been undertaken as a promising alternative to the previously discussed ML models. DNNs, with their ability to learn complex patterns and representations from large datasets, offer a potential advantage in recognizing detailed fault patterns that traditional ML models might overlook.

### Deep neural network architecture (DNN)

**Fig. 7:**
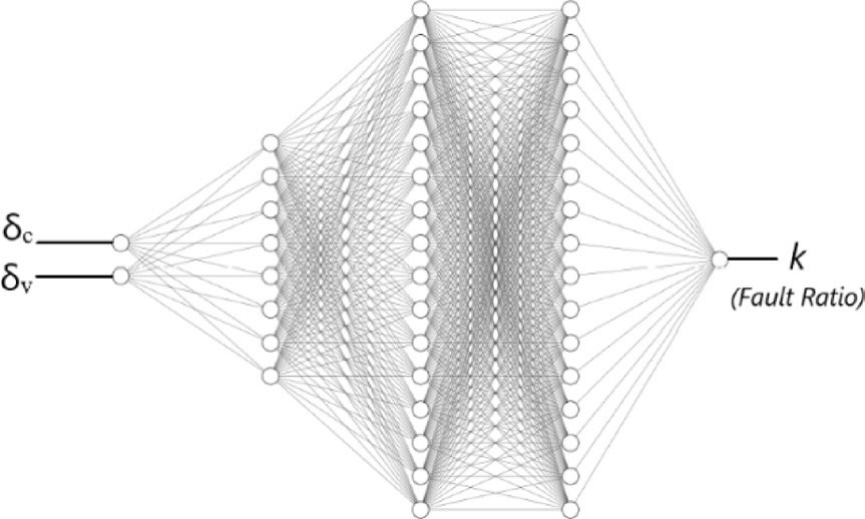
Deep neural network architecture.

The architecture of the proposed DNN for the diagnosis system, as shown in Fig. 7, is detailed below.

*Input layer* consists of 2 neurons accepting δ_c_ and δ_v_ in their respective complex formats, as derived in the *Fault Features* section. A complex-split mechanism is used to process these inputs effectively.

*1st hidden layer* A fully connected dense layer with 8 neurons, using the hyperbolic tangent (tanh) activation function. This layer begins the feature extraction process by introducing non-linearity and transforming the input into a more abstract representation.

*2nd hidden layer* Another fully connected layer, expanded to 16 neurons and also using tanh activation. With its deeper structure, it captures more complex patterns, enhancing the model’s ability to generalize.

*3rd hidden layer* A fully connected layer with 16 neurons, utilizing the rectified linear unit (ReLU) activation. ReLU helps mitigate the vanishing gradient problem, improves training efficiency, and promotes sparsity for better fault discrimination.

*Output layer* is the final fully connected dense layer of the neural network, consisting of 1 neuron. It is responsible for producing the network’s output based on the learned representations from the previous layers. The output layer uses the sigmoid activation function, which maps the output to a probability range between 0 and 1. In the context of fault diagnosis, this setup allows the network to provide predictions representing fault severity (0: Represents no fault detected) and (1: Represents 100% fault ratio). The sigmoid activation function ensures that the network’s estimation is bounded within this interpretable range.

### Training and testing of DNN model

The same training dataset previously used for ML models is considered for training the DNN model. To develop a robust predictive model, 80% of the dataset (17,775 cases) was used for training, while the remaining 20% (4443 cases) was reserved for training validation. The data partitioning was done randomly to ensure the model’s performance could be accurately assessed across a diverse range of data points, minimizing biases and ensuring reliability in real-world applications.

The DNN model was implemented using the *MATLAB Deep Learning Toolbox*, which provides a comprehensive environment for designing, training, and validating deep learning models. This toolbox is well-suited for handling large datasets and complex model architectures. Training of the DNN model was performed using the Adam optimization algorithm, known for its efficiency in handling large datasets and complex models within the MATLAB Deep Learning Toolbox. The training process was structured to span a maximum of 100 epochs, with convergence typically achieved within just 20 epochs, totaling 35,050 iterations. The initial learning rate was set at 0.01, with a learning rate reduction mechanism applied after each epoch. This adaptive learning rate strategy involves reducing the learning rate sequentially, which refines the adjustments made by the optimizer as training progresses. This method of dynamically adjusting the learning rate is critical for optimizing the training process and enhancing the model’s performance in fault detection tasks. A batch size of 64 was used, which is optimal for balancing the computational load and effective handling of the dataset size. The categorical cross-entropy loss function was employed, appropriate for the multi-class classification framework of our study.

**Fig. 8:**
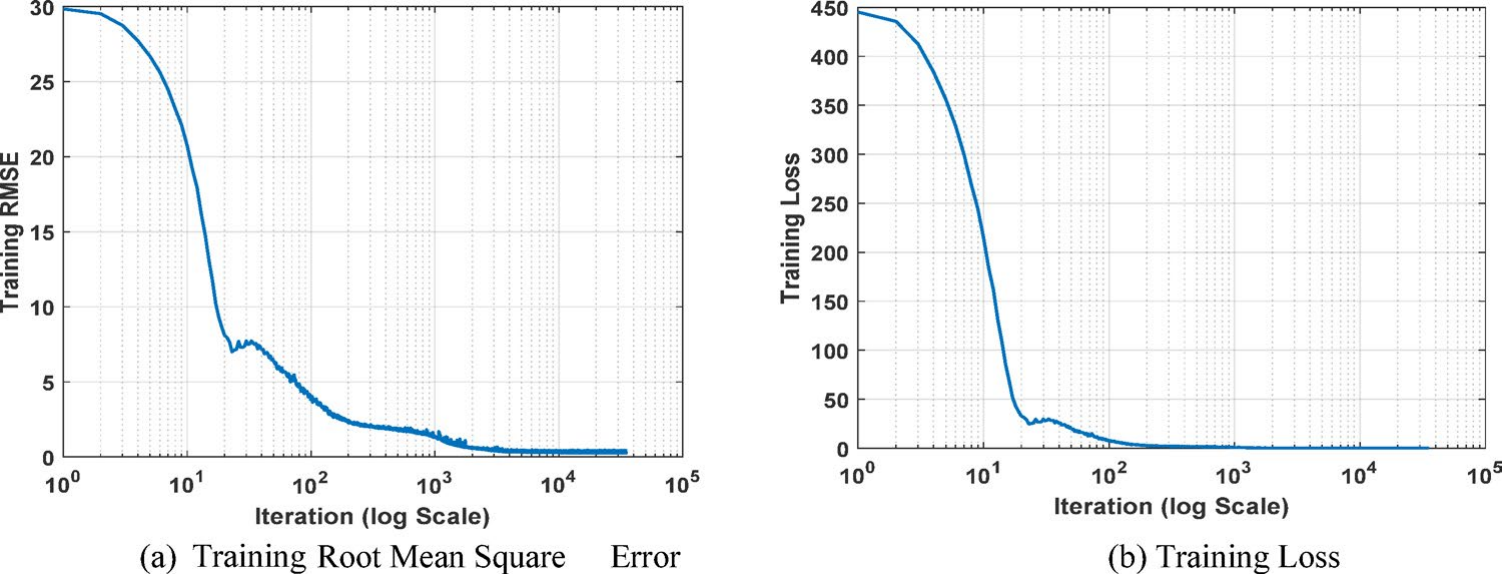
DNN training performance.

As depicted from Fig. 8, the training process demonstrates rapid initial learning, as evidenced by the sharp decline in RMSE and loss within the first few iterations. Figure 8a shows the training RMSE plotted against the number of iterations on a logarithmic scale. The RMSE decreases from a high value of approximately 30 to less than 5 within the first 1000 iterations and continues to decline more gradually thereafter. By the end of training, the RMSE stabilizes at a final validation value of 0.32779, indicating high model accuracy. Similarly, Fig. 8b presents the training loss plotted against the number of iterations on a logarithmic scale. The loss metric shows a steep drop from around 400 to near-zero levels, with a final loss of 0.054, reflecting the optimizer’s effectiveness in minimizing error. This configuration balances rapid initial learning with fine-tuned adjustments, ultimately leading to a highly accurate and effective model.

## Simulation and experimental validation

### Simulation validation tests

To validate the proposed fault detection approach, the AI models in the study were further tested on a separate test dataset to evaluate their performance under new conditions. This test dataset included fault ratios ranging from 1 to 49% in 2% increments, ensuring a comprehensive evaluation across both low and high fault ratios. Additionally, the dataset incorporated scenarios with USV conditions, which were not included in the training data. These conditions involved phase C voltage reductions ranging from 0.2 to 2.8% in increments of 0.2%, and for each reduction in phase C, corresponding reductions in phase B were also applied. The same performance metrics used for cross-validation are considered for test dataset. DNN performance metrics are also calculated, and all the obtained results are listed in Table 3.

**Table 3:** Performance metrics for test data.

Model	MSE	RMSE	MAE
DNN	0.0642	0.2533	0.1729
Gradient boosting	0.201	0.448	0.373
kNN	0.7	0.836	0.728
Random forest	0.775	0.88	0.804
AdaBoost	0.966	0.983	0.969
Tree	1.146	1.07	0.978
Linear regression	29.476	5.429	4.878
PLS	31.05	5.572	4.947
Stochastic gradient descent	37.558	6.128	5.568
SVM	338.514	18.399	15.03

From Table 3, DNN has emerged as the top AI performer delivering a noteworthy performance with an MSE of 0.0642, RMSE of 0.2533, and MAE of 0.1729, indicating it may offer a promising alternative with lower error metrics than ML models. As for ML models, the results on the test data generally mirrored the trends observed during cross-validation. Gradient Boosting maintained its leading position as second to DNN, with minimal increases in MSE and RMSE, demonstrating its strong generalization capability. Both kNN and RF continued to perform well, although their errors slightly increased compared to the cross-validation results, which is typical when applying models to new data. However, AdaBoost and decision tree exhibited larger increases in error metrics, indicating these models may be more sensitive to variations in test data. Linear regression, SGD, and PLS performed consistently with their cross-validation results, further confirming their limitations in dealing with the complexity of the fault data. SVM remained the poorest performer.

**Fig. 9:**
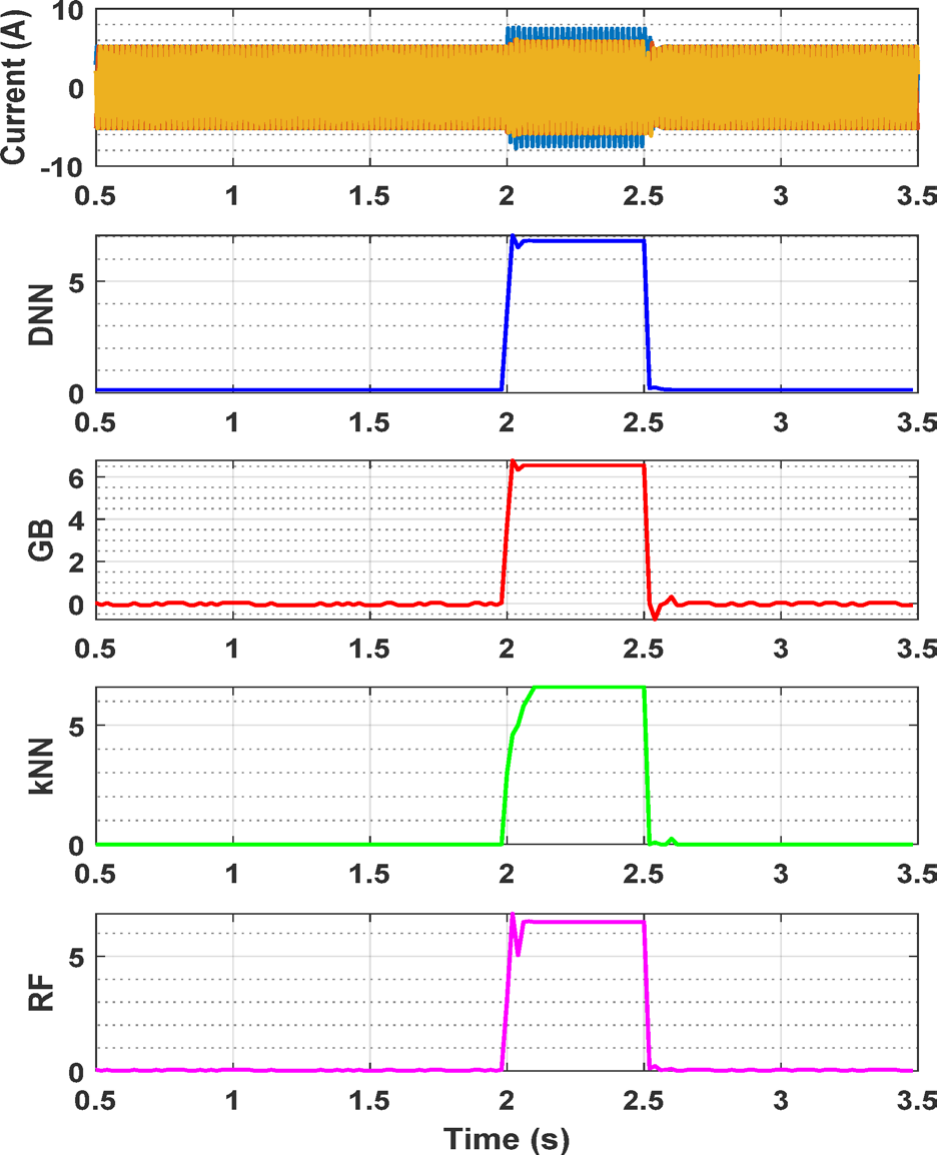
Fault ratio estimation in percentage over time for 7% fault ratio in phase A.

Figure 9 presents the fault detection performance of 4 top AI models over time for a specific fault case: a 7% fault ratio in phase A with a balanced supply voltage. This case is used as a sample to demonstrate the effectiveness and reliability of the fault detection approach in real time application. The results depicted in Fig. 9 show that all 4 models successfully detect the fault in phase A with 6.824%, 6.549%, 6.599% and 6.495% fault ratios for DNN, Gradient Boosting, kNN and RF, respectively. The top obtained detection error is about 0.176% for DNN which remains consistent over time, indicating the robustness of the Model.

**Fig. 10:**
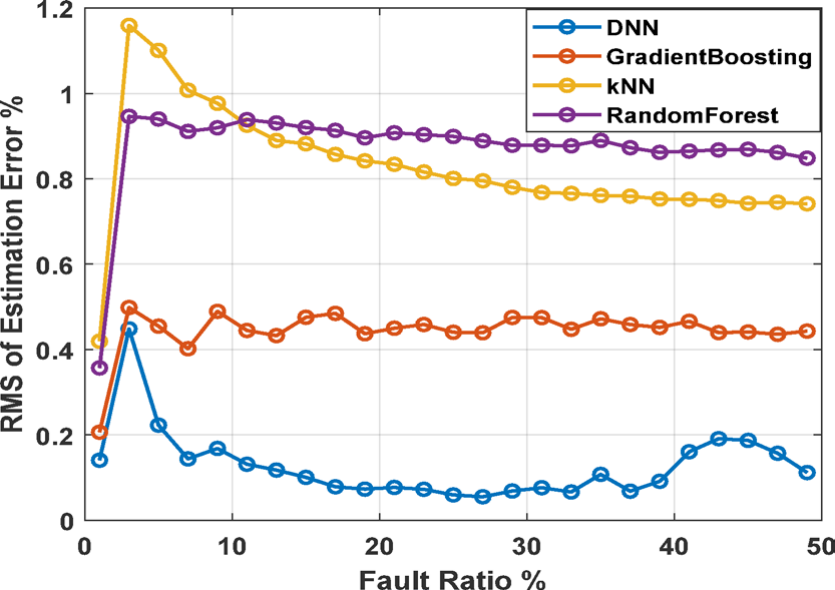
RMS of estimation error for various fault ratios and supply voltage unbalance conditions.

The estimation error analysis, as depicted in Fig. 10, provides a detailed comparison for RMS of estimation error across fault ratios ranging from 1 to 49% for top four AI models. The analysis reveals that DNN consistently achieves the lowest RMS estimation error across almost all fault ratios, indicating its superior accuracy and robustness in fault prediction. Gradient Boosting also performs well, with slightly higher errors than DNN but maintaining low variability, especially at lower fault ratios. RF shows a more noticeable increase in estimation error as the fault ratio rises, particularly after 10%, suggesting that while it performs adequately, it may be less stable under more severe fault conditions. kNN demonstrates a clear upward trend in estimation error with increasing fault ratios, indicating that its performance deteriorates as the severity of the fault increases.

**Fig. 11:**
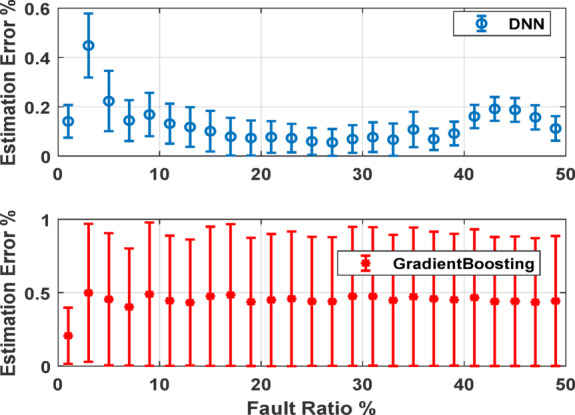
Error bars across USV conditions for DNN and GB against fault ratios.

Figure 11 presents a detailed analysis of the estimation error for the two top performing AI models with error bars representing the variability in model predictions under supply voltage unbalance conditions. The top chart shows the DNN model’s estimation error with a relatively low and consistent error margin across most fault ratios. The error bars indicate low variability, especially for fault ratios between 10 and 40%, underscoring the model’s stability and accuracy in prediction. The bottom chart illustrates the Gradient Boosting model’s estimation error. While this model also maintains a consistent error level across different fault ratios, the error bars reveal significantly higher variability compared to the DNN model. This suggests that while Gradient Boosting can produce accurate predictions, its performance is less stable, with a higher degree of uncertainty in its estimations.

As concluded, the DNN model stands out with its consistently low errors and stability across a wide range of fault ratios, while Gradient Boosting also shows commendable performance with minimal error fluctuations. In contrast, kNN and RF, despite being effective at lower fault ratios, exhibit increased errors and variability, particularly as the fault ratio becomes more significant. This suggests that DNN and Gradient Boosting are more reliable for handling a broader spectrum of fault conditions, with DNN being the most robust and accurate model in this comparison.

### Experimental validation tests

**Fig. 12:**
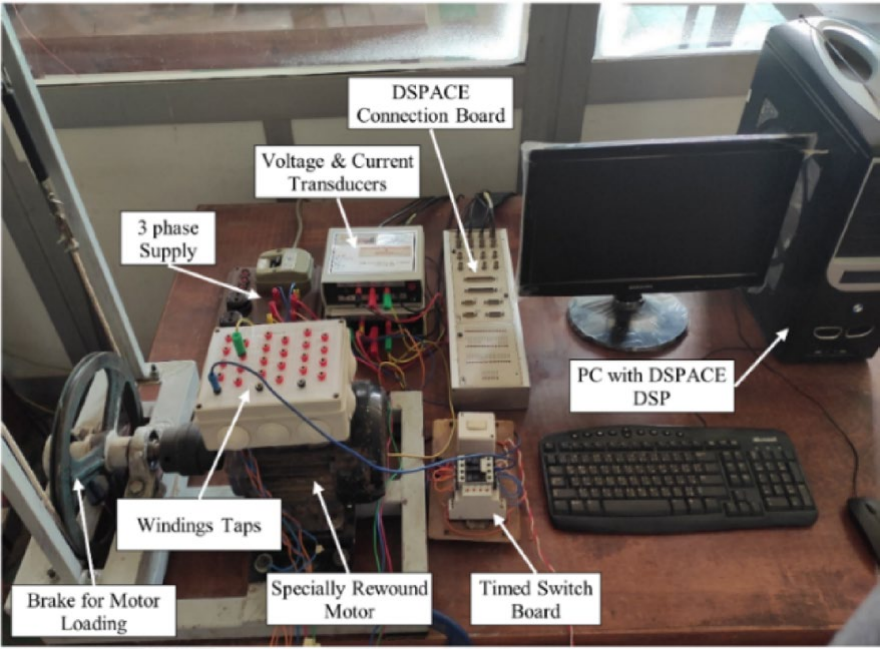
Fully constructed experimental test bench system.

To validate the effectiveness of the developed diagnostic approach, an experimental investigation was conducted using the test-bench described in^40^. Figure 12 illustrates the experimental setup. The test-bench includes a power supply voltage source connected to the terminals of the three star-coupled stator phases of IM. A rewound three-phase IM, matching the parameters of the IM model presented in Table 1, is employed. The stator features six coils per phase, with each phase having 12 taps used to mimic ITFs.

To accurately simulate fault conditions, a technique involving the short-circuiting of different taps has been implemented. This is facilitated by an external switchboard enabling control over fault timing and monitoring of fault current during the experiments. A mechanical braking system is used to apply loads to the motor. Voltage and current measurements are taken using LEM LV 25-P and LEM LA 25-NP transducers, which are connected to a DSPACE DS1104 DSP with an I/O board CP1104. To ensure the accuracy and reliability of the measurements, all devices underwent periodic calibration checks. Additionally, the data acquisition system was recalibrated in the laboratory using reference voltage and current waveforms before starting the experiments.

To ensure comprehensive evaluation of the proposed diagnostic system, the experimental testing was designed to span a wide range of real-world operating conditions. These include multiple fault severities ranging from 8.33 to 58.33%, unbalanced supply voltages from 3 to 10%, and various mechanical load levels applied through a braking system. Each condition was tested using repeated measurements, enabling assessment of both diagnostic accuracy and robustness. This experimental diversity was intended to simulate the kinds of challenges commonly encountered in industrial environments and to validate the system’s adaptability under realistic fault scenarios.

The practical implementation of the AI-based diagnostic system aligns with the architecture outlined in the Deployment Phase. During the experimental evaluation, three-phase voltage and current signals were captured from the motor terminals using LEM transducers and sampled via the DSPACE DS1104 DSP card. These raw signals were transferred in real time to the host PC, where further processing was handled in MATLAB. For each electrical cycle, a recursive DFT was applied to compute the voltage and current phasors at the power frequency (50 Hz). Based on these phasors, the symmetrical components of voltage and current were calculated to obtain fault features (δ_v_ and δ_c_). These two features were then used as inputs to the previously trained DNN model, implemented in MATLAB. The DNN produced a corresponding fault ratio estimation, and the process was repeated for the subsequent electrical cycles.

**Fig. 13:**
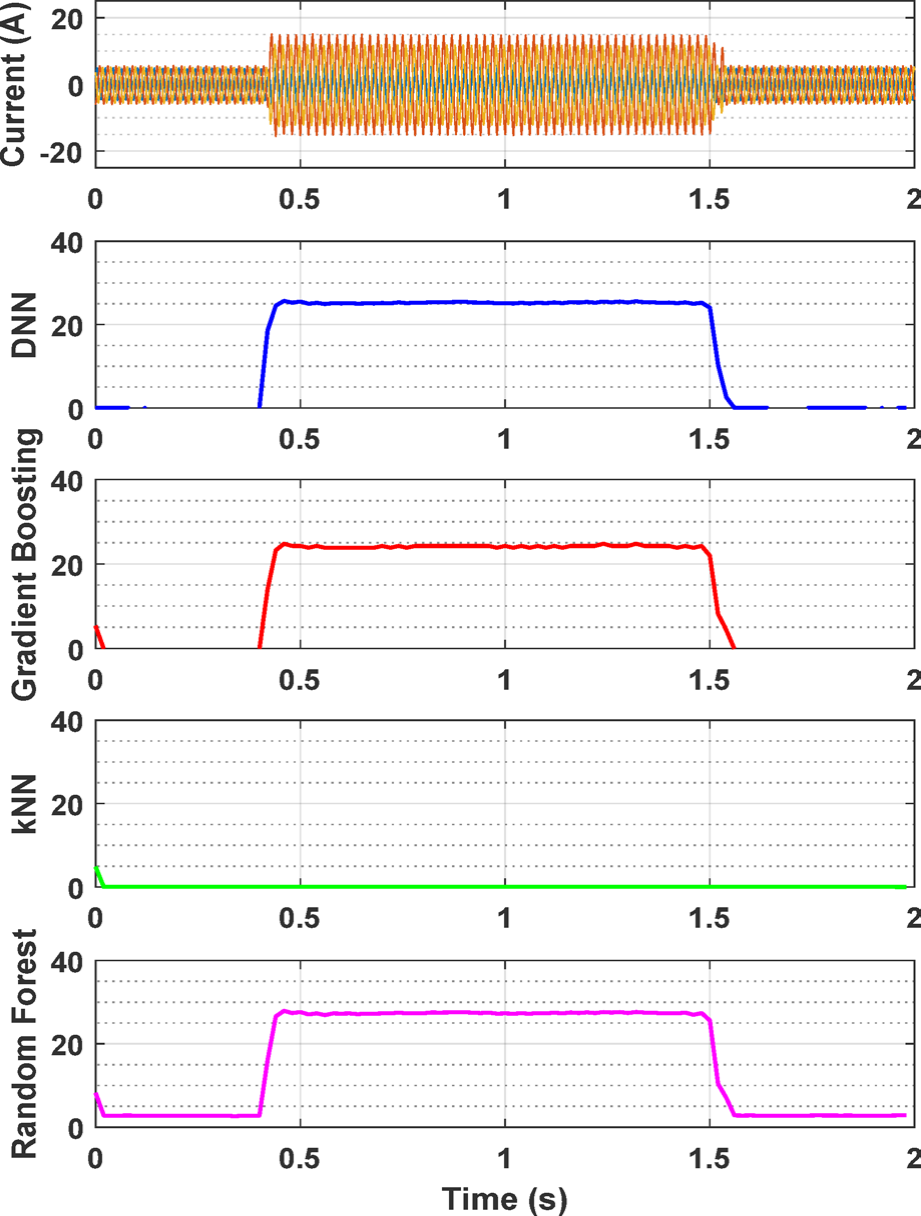
Current waveform and fault ratio estimation over time for 25% fault ratio.

**Fig. 14:**
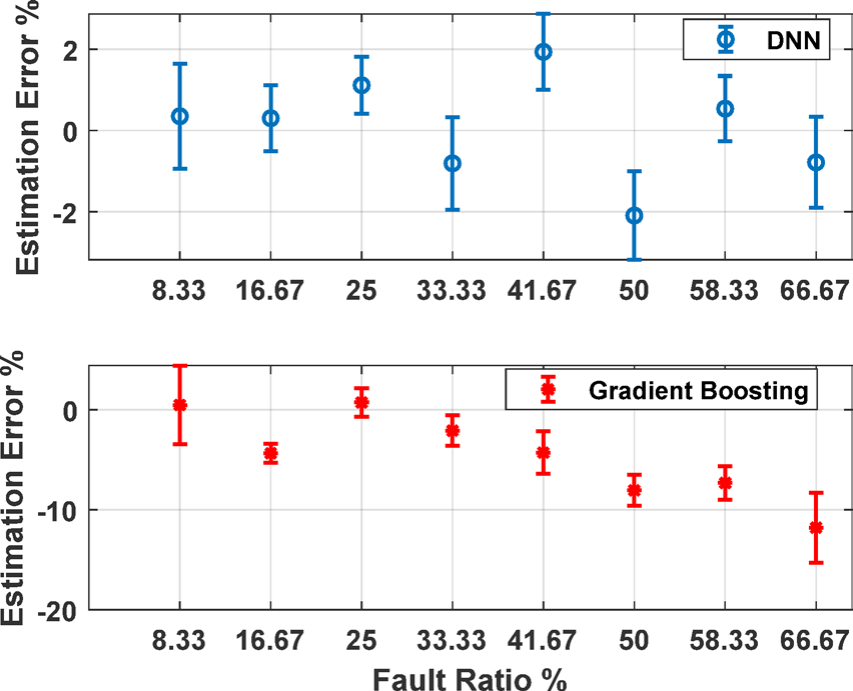
Experimental estimation error bars for various fault ratios and unbalance conditions in phase C.

The results from the experimental setup are shown in Figs. 13 and 14. Figure 13 illustrates the current waveform and top four AI model fault ratio estimations over time for a 25% fault ratio, demonstrating the system’s response under a specific fault condition. This figure highlights how the current increases significantly when the fault is introduced and returns to normal once the fault is cleared. Among the models, DNN provided the closest estimate, with a value of 25.0897%, demonstrating the highest accuracy detecting the fault and estimating its severity. Gradient boosting also performed well, producing an estimate of 24.2521%, slightly underestimating but still offering a reasonable prediction. The RF model, while providing a generally accurate estimate, deviated further with a value of 27.2441%, indicating less precision compared to DNN and gradient boosting. The kNN model failed to detect the fault and to produce a meaningful estimate, as evidenced by its flat response in the plot, suggesting it is not well-suited for this application.

Figure 14 summarizes the estimation errors for various fault ratios and USV conditions. Specifically, it shows the results for balanced voltage conditions along with 3%, 5%, 7%, and 10% reductions in phase C voltage. The DNN model’s performance is highlighted in the top plot, where the estimation error remains within approximately ± 2.5% demonstrating the model’s accuracy and robustness in fault detection. The Gradient boosting model’s performance is shown in the bottom plot, where it consistently exhibits a negative bias in its estimation errors, particularly at higher fault ratios, with errors reaching as much as − 15% under more significant USV conditions.

A critical aspect of the system’s validation involved examining the impact of motor loading on the fault detection capabilities. Despite varying the load applied through a mechanical braking system, the analysis revealed that the system’s performance remained unaffected. This consistency was mirrored in the simulation results where δ_c_ was shown to remain stable across different loading conditions, as illustrated in Fig. 5.

**Fig. 15:**
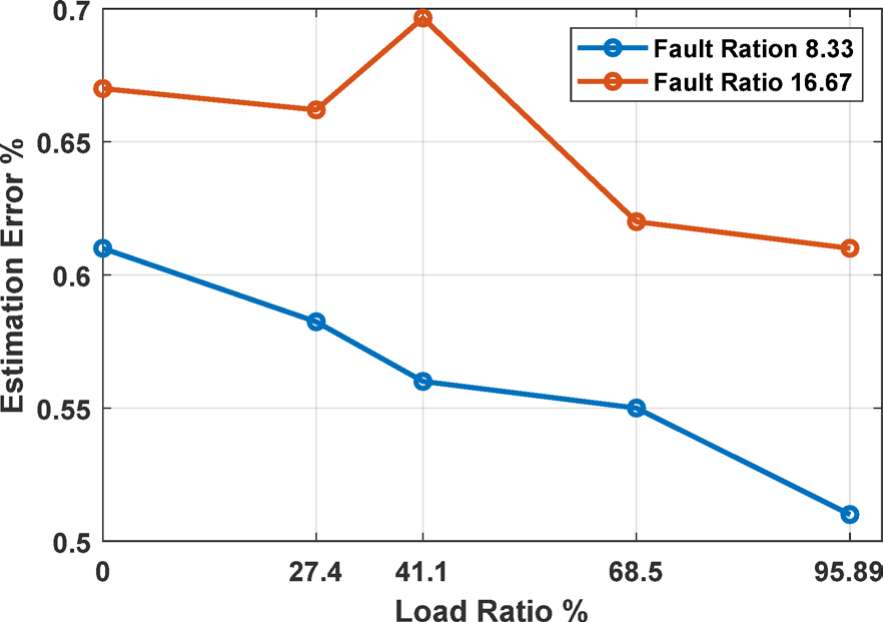
Impact of load ratio on estimation errors for different fault ratios.

To provide a comprehensive view of how motor loads affect fault estimation accuracy, the estimation errors across varying load ratios were analyzed, particularly focusing on two specific fault ratios of 8.33% and 16.67%. As depicted in Fig. 15, changes in load ratio were found to have a minimal impact on the estimation errors, confirming the robustness of the fault detection system under varying operational conditions. While slight fluctuations in estimation accuracy were observed, these did not significantly affect the overall performance of the DNN model. Given that the experimental outcomes confirm these simulation predictions, showing negligible effects on the DNN’s performance across different load conditions.

The experimental results aligned closely with the simulation outcomes, confirming that the DNN model is highly reliable under real-world conditions. The DNN model maintains low estimation errors across all tested conditions, showing its superior capability in handling varying ITFs ratios and USV. In contrast, the Gradient Boosting model, while still effective, shows greater sensitivity to ITFs ratios, particularly under conditions of higher USV, where the estimation errors are notably larger.

**Fig. 16:**
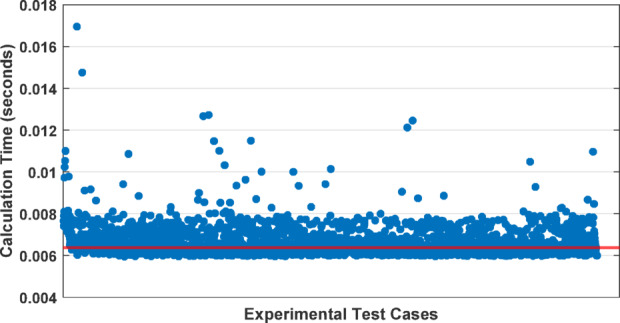
Distribution of computation times for DNN-based ITF detection across experimental cases.

To further validate the effectiveness of the DNN model, we conducted an analysis of computational efficiency, specifically focusing on the processing times required for fault detection. This fault detection method employs the trained DNN and operates on a per-window basis. In this context, each ‘window’ refers to the time span of one electrical cycle, within which the entire detection process is executed. The timing for detection procedure includes several stages. Firstly, the sequence components of both voltage and current are calculated. Following this, δ_c_ and δ_v_ are computed, which are used as inputs to DNN. Subsequently, the trained DNN model analyzes these coefficients to identify and quantify the fault ratio, thereby estimating the severity of any detected faults. This process is implemented using MATLAB R2022b and the Deep Learning Toolbox on a system equipped with an Intel Core i7-3610QM processor and 8 GB of RAM. Figure 16 shows the distribution of processing times for each recorded experimental window, underscoring the system’s efficiency. Each data point on the scatter plot represents the computation time for processing a single window, with the median processing time notably low at 6.4 ms as represented by red line. This low latency confirms the system’s suitability for real-time fault detection, which is crucial in industrial environments where delays in fault detection can lead to significant operational disruptions and safety hazards. The low-dimensional feature space and efficient architecture of the model also support future deployment on embedded systems such as DSPs or FPGAs for fully autonomous field implementation.

The proposed DNN-based ITFs detection system demonstrates high diagnostic accuracy and computational efficiency, making it well-suited for real-time industrial monitoring. Its ability to detect faults reliably across varying conditions, handling fault ratios from 8.33 to 58.33% and voltage unbalance up to 10%, supports its robustness and practical applicability. Validation in both simulation and experimental conditions confirms the model’s effectiveness in enhancing preventive maintenance, reducing downtime, and enabling intelligent, automated fault diagnostics in induction motors.

While DNN approach demonstrated strong diagnostic performance, it is essential to consider typical limitations associated with deep learning models; generalization ability, interpretability, and data efficiency. In the present study, DNN was trained exclusively on simulated data generated from a high-fidelity motor model previously validated in^40^. The model achieved high accuracy when tested on experimental data, indicating strong generalization across different data domains. This performance can be attributed to two key factors. First, the simulation model accurately replicates realistic motor behavior under ITF conditions. Second, the use of two abstract yet physically grounded features, δ_c_ and δ_v_, enables consistent capture of fault-relevant characteristics in both simulated and experimental scenarios. Regarding interpretability, although DNNs are inherently considered black-box models, the use of a minimal and physically interpretable input set improves transparency to a certain extent, as δ_c_ and δ_v_ are directly derived from terminal electrical quantities. Nevertheless, full explainability remains a limitation. With respect to data efficiency, the reliance on simulated data addresses the typical data volume requirements of DNNs. However, this approach remains viable only when real-world operating conditions can be accurately modeled.

As the validation results clearly demonstrate the practical effectiveness of the proposed approach, it is also important to position these results in the broader context of existing research. Many previously published methods show good performance in controlled settings yet fall short under industrial constraints such as USV, variable loading, or the need for additional sensors. In the following section, a comparative analysis is presented to critically evaluate the capabilities and limitations of prior approaches, highlighting how our method addresses long-standing gaps in the field.

## Comparative analysis of ITFs detection methods

The ITFs diagnosis approach developed in this study utilizes innovative fault features; δ_c_ and δ_v_, which are uniquely engineered to effectively capture the effects associated with ITFs in IMs from their terminal measurements. These features are characterized by their robustness against motor loading conditions, ensuring that the system’s fault detection capabilities remain stable across various operational states. The fault features are extracted from an accurately simulated induction motor model, reflecting realistic motor behaviors under ITFs conditions.

The employment of DNN reflected on the efficacy of this diagnostic approach due to its unique ability to capture complex relations in input data. Despite being trained solely on simulation data derived from the motor model, the DNN exhibits exceptional efficiency in detecting and estimating the severity of ITFs from direct experimental measurements. This remarkable capability highlights the proposed approach’s ability to generalize from simulation training environments to real-world applications, without requiring prior training on experimental data.

The following points aim to highlight the unique aspects of the proposed ITF diagnostic approach and its advantages over previous studies in the field of ITFs and USV diagnosis in IMs. A detailed comparative analysis is provided, with findings summarized in Table 4. This comparison underscores how the proposed methodology advances beyond the current studies, offering significant improvements in fault detection accuracy and operational robustness.

- *ITF detection range* The proposed approach excels in its extensive coverage of fault severities, detecting minor faults starting from as low as 1% and extending to severe faults up to 50%. This broad detection range is significantly more comprehensive compared to other studies in the field, providing a marked improvement in diagnostic capabilities. For instance, study^48^ is limited to detecting faults in a smaller range from 1 to 10%, while reference^24^ covers up to 27%. Notably, reference^49^ only starts detecting faults at 10%, missing critical early-stage fault identification.
- *USV accounting* Unlike several studies such as in^3,15,36,49,50^ which do not account for USV, the proposed approach integrates the diagnosis of both ITFs and USV, similar to the approach seen in^24,48^. This integration is crucial as it allows for accurate fault detection under varied power supply conditions, as reflected in both simulation and experimental results.
- *Robustness against load variation* Many studies have overlooked the effects of load variations on fault diagnostics, including those referenced in^35,51^. In contrast, others, such as^3,24,36,48–50^, have acknowledged and examined the impact of load variations. The proposed approach excels in maintaining robust performance irrespective of load conditions. This ensures reliable fault detection even when the induction motor operates under diverse loading scenarios, thereby enhancing the system’s applicability for industrial uses.
- *Cost-effectiveness* The proposed diagnostic approach is designed to be cost-effective, requiring no additional sensors for operation. This contrasts with approaches such as those mentioned in^24^, where expensive torque sensors are necessary, thus increasing the overall cost of the diagnostic setup.
- *High accuracy* The diagnostic approach demonstrates a high level of accuracy, with experimental validations consistently showing an accuracy of estimation not less than 98%. This level of performance significantly surpasses that of many other models discussed in the literature. For instance, studies such as^35,49^ often lack detailed statistical analysis when discussing reliability. In contrast, accuracies in^3,24,36,50^ range between 45 and 99%, while another study^48^ reports accuracies ranging from 95 to 100%. The consistently high accuracy of this work ensures that the system can reliably identify and quantify fault conditions.
- *Computational efficiency* Moreover, the proposed diagnostic approach is optimized for low computational demands, enabling high-speed fault detection suitable for real-time applications. The system’s ability to rapidly process and analyze data ensures timely and accurate fault diagnosis, making it an invaluable tool for continuous monitoring and preventive maintenance strategies.

**Table 4:** Comparative analysis for ITFs diagnosis approaches.

	Load variation	USV variation	ITFs detection	ITFs severity	Additional sensor	Detection range	Accuracy
^3^	✓	✗	✓	✓	✗	2–10%	45–98%
^24^	✓	✗	✓	✓	✓	Up to 27%	88–99%
^35^	✗	✗	✓	✓	✗	2–25%	N. S.
^36^	✓	✓	✓	✓	✗	N. S.	93–99%
^48^	✓	✓	✓	✓	✗	1–10%	95–100%
^49^	✓	✗	✓	✓	✗	From 10%	N. S.
^50^	✓	✗	✓	✓	✗	N. S.	95%
^51^	✗	✓	✓	✗	✗	N. S.	N. S.
This work	✓	✓	✓	✓	✗	1–50%	98–100%

## Conclusion

ITFs pose significant challenges to the operation of IMs, which serve as critical components in a wide range of industrial applications. In response to this challenge, this study proposed a novel AI-based diagnostic approach for accurate detection and severity estimation of ITFs, including under USV conditions, often overlooked in existing methodologies.

The proposed system relies exclusively on voltage and current measurements from motor terminals, eliminating the need for additional sensors and reducing implementation costs. Two physically meaningful features were engineered: the complex current unbalance coefficient (δ_c_) and the complex voltage unbalance coefficient (δ_v_). The coefficient δ_c_, derived from the symmetrical components of motor current phasors, showed a strong correlation with ITF severity while remaining unaffected by variations in motor load, validating its robustness as a primary diagnostic feature. To distinguish between current imbalance caused by ITFs and that induced by USV, δ_v_ was defined using the symmetrical components of the supply voltage. Together, these two features enable reliable differentiation between fault-induced and supply-induced anomalies.

Multiple ML models and a DNN were trained using a comprehensive dataset generated from high-fidelity SIMULINK simulations of IMs under varying fault and voltage conditions. Among the tested models, DNN emerged as the most effective, demonstrating superior generalization and accuracy. The system was further validated through extensive experimental testing using a custom-built test bench, simulating a wide range of realistic operating conditions including fault severities (8.33–58.33%), voltage unbalance (up to 10%), and variable mechanical loading. DNN maintained estimation errors within ± 2.5%, despite potential measurement noise and model uncertainties.

This high diagnostic accuracy, coupled with a fast-processing time of just 6.4 ms per detection cycle, affirms the system’s suitability for real-time industrial deployment. Its computational efficiency and low-dimensional feature space further support future implementation on embedded systems such as DSPs and FPGAs for edge-based, autonomous diagnostics.

Compared to prior research, this approach offers several key advantages: a wider detection range (1–50% ITFs), load-invariant performance, resilience to USV-induced false alarms, sensorless implementation, and consistently high fault estimation accuracy (98–100%). These contributions mark a significant advancement in the field of intelligent motor diagnostics.

This research demonstrates that combining advanced signal processing, robust feature engineering, and deep learning yields a powerful, efficient, and scalable solution for ITF diagnosis in IMs. Future work may focus on enhancing model interpretability, extending the framework to other motor fault types, and deploying the system on embedded hardware for fully autonomous, real-time industrial monitoring.

## Data Availability

The datasets generated and/or analyzed during the current study are available from the corresponding author on reasonable request. For further inquiries, please contact Wagdy M. Khater at Wagdy.Khater@sh-eng.menofia.edu.eg
